# MR Brain Screening in ADPKD Patients: To Screen or not to Screen?

**DOI:** 10.1007/s00062-021-01050-0

**Published:** 2021-09-29

**Authors:** I. Capelli, M. Zoli, M. Righini, L. Faccioli, V. Aiello, L. Spinardi, D. Gori, F. Friso, A. Rustici, C. Bortolotti, C. Graziano, V. Mantovani, N. Sciascia, D. Mazzatenta, M. Seri, M. Pastore Trossello, G. La Manna

**Affiliations:** 1 Nephrology, Dialysis and Renal Transplant Unit, IRCCS – Azienda Ospedaliero Universitaria di Bologna, Alma Mater Studiorum, University of Bologna, Bologna, Italy; 2 Department of Neurosurgery, IRCCS Istituto delle Scienze Neurologiche di Bologna, Bologna, Italy; 3 Department of Biomedical and Neuromotor Sciences (DIBINEM), University of Bologna, Bologna, Italy; 4 Department of Neuroradiology, IRCCS Istituto delle Scienze Neurologiche di Bologna, Bologna, Italy; 5 Department of Experimental, Diagnostic and Specialty Medicine (DIMES), University of Bologna, Bologna, Italy; 6 Medical Genetics Unit, Sant’Orsola-Malpighi University Hospital, Department of Medical and Surgical Sciences, University of Bologna, Bologna, Italy; 7 Radiology Unit, S. Orsola-Malpighi University Hospital, Bologna, Italy; 8 Center for Applied Biomedical Research (CRBA), University of Bologna, Bologna, Italy

**Keywords:** Polycystic disease, Intracranial aneurysms, Acute complications screening, Genetic nephropathies, Arachnoid cysts

## Abstract

**Background:**

Adult polycystic kidney disease (ADPKD) still represents a major cause of renal failure and intracranial aneurisms (IA) have a higher prevalence in ADPKD than in the general population. Current guidelines suggest performing brain MRI only in the subjects with a positive familiar history of IAs or subarachnoid hemorrhage (SAH). This is a retrospective case-control analysis to evaluate the usefulness of a MR screening program in ADPKD patients.

**Methods:**

We retrospectively analyzed all ADPKD patients followed in our outpatient clinic between 2016 and 2019 who underwent a brain MRI screening. We evaluated the presence of IAs and others brain abnormalities and compared our results with a non-ADPKD population (*n* = 300). We performed univariate and multivariate regression analysis to evaluate if general and demographic features, laboratory findings, clinical parameters and genetic test results correlated with IAs or other brain abnormalities presence.

**Results:**

Among the patients evaluated 17 out of 156 (13.6%) ADPKD patients had IAs, compared to 16 out of 300 (5.3%) non-ADPKD controls (*p* < 0.005). Considering ADPKD patients presenting IAs, 12 (70.6%) had no family history for IAs or SAH. Genetic analysis was available for 97 patients: in the sub-population with IAs, 13 (76.5%) presented a PKD1 mutation and none a PKD2 mutation. We found that arachnoid cysts (AC) (*p* < 0.001) and arterial anatomical variants (*p* < 0.04) were significantly more frequent in ADPKD patients.

**Conclusion:**

In our population ADPKD patients showed a higher prevalence of IAs, AC and arterial variants compared to non-ADPKD. Most of the IAs were found in patients presenting a PKD1 mutation. We found a significant number of alterations even in those patients without a family history of IAs or SAH. The practice of submitting only patients with familial IAs or kidney transplantation candidates to MRI scan should be re-evaluated.

## Introduction

Adult polycystic kidney disease (ADPKD) is one of the most common life-threatening genetic disorders caused by single gene mutations, presenting a worldwide prevalence of 1:400–1000 live births. About 50% of adult ADPKD patients will require dialysis or kidney transplantation within their 6th decade [1, 2].

The prognosis is often worsened by extrarenal manifestations of the disease, such as the rupture of intracranial aneurisms (IAs). Although several authors have demonstrated that ADPKD patients are at higher risk of developing IAs (estimated prevalence: 9–12% of all ADPKD patients vs. 2–3% of the general population), the underlying reasons for this association are still unknown [1, 2]. Particularly, the mechanism by which specific PKD mutations predispose to a vascular phenotype remains unclear; moreover, the prognostic factors associated with a greater risk of IAs development and/or rupture are underinvestigated in ADPKD patients [3, 4].

The early detection of IAs is highly desirable; indeed, it may allow a timely treatment of the brain vascular lesion in elective and more favorable conditions, avoiding the dramatic risks associated to subarachnoid hemorrhage (SAH), such as brain ischemia for vasospasm, hydrocephalus or intraparenchymal hematomas [5, 6]. At the state of art, the sole evidence is that ADPKD patients with a positive family history of SAH or IAs should be considered at increased risk of developing an IAs [3, 4], therefore current guidelines support the neuroradiological screening of ADPKD patients with MR angiogram (MRA) only in these patients [7]. Conversely, recent relevant studies suggested that, also in absence of a SAH familiar history, ADPKD patients are more susceptible to IAs, with about a fivefold increase in the rupture rate of vascular lesions compared to general population [5].

The vast majority of studies have focused on this association between ADPKD and IAs; however, the latter are not the only pathological anomaly of the brain that can be detected with an MR study in ADPKD patients. Indeed, some authors have suggested that these cases are more susceptible to arachnoid cysts, or other vascular malformations, or arterial anatomical abnormalities and variants [8, 9].

This study is a retrospective case-control analysis of pathological or nonpathological brain findings through magnetic resonance (MR) in the population of ADPKD patients and compared them to a non-ADPKD population, to assess what conditions are more relevantly associated with ADPKD and require a particular care in clinical management. Moreover, other possible factors associated to these brain pathological and non-pathological conditions have been analyzed, with the aim of identifying patients at higher risk, feasibility, and requiring a more careful and strict screening.

## Methods

We retrospectively analyzed, according to the unified ultrasonographic criteria, all ADPKD patients followed in our outpatient clinic between 2016 and 2019 who had undergone MRI and MRA (1.5 T) screening for brain pathologies [11]. The MR study was managed on General Electric (Signa HDxt, GE Healthcare, Milwaukee, WI, USA) 1.5 T through sagittal T1 FSE, axial FLAIR, coronal T2 FSE, DWI, axial T2 *, Angio-RM 3D TOF sequences and on Philips (Ingenia, Philips Healthcare, Best, The Netherlands) 1.5 T spare parts through sagittal 3D T1 SPIR sequences, axial FLAIR SPIR, coronal T2 TSE, DWI, SWI, Angio-RM 3D TOF. axial T1 FSE, coronal T1 fat-sat and coronal T1 Dixon sequences have been added in studies where contrast medium completion is required. The contrast medium used was Gadovist® (Bayer HealthCare, Toronto, Canada), at a dosage of 0.1 mmol/kg.

At the time of the outpatient observation, we had collected demographic information (sex and age), renal function indices (serum creatinine, eGFR values, stage of CKD), clinical parameters (presence/absence of arterial hypertension, family history of ADPKD or family history of hemorrhagic stroke and SAH, smoking habit) and the results of genetic tests. Arterial hypertension was considered as blood pressure > 140/90 mm Hg or positive history of antihypertensive therapy. Before performing the genetic test, patients signed an informed consent. Family history was considered positive in cases of diagnosis of ADPKD or IA/SAH in a first-degree relative.

At brain MR, the presence of IAs and their features (location, maximum diameter and dome-to-neck ratio) has been evaluated. The presence of other vascular lesions (arterovenous malformations or cavernomas), the presence of arterial anatomical variations (i.e. fetal variant of posterior communicating artery, PComA, arterial duplications, fenestrations or azygous variants) or arachnoid cysts (AC) have been included in the same examination. The PHASES score was calculated for every IA in both populations [26]. The images were reviewed by two independent readers (neuroradiologists with 5 and 13 years of experience, respectively) from 2 different institutions, blinded to the clinical data but aware of patients aneurysm locations, independently reviewed the images. They were asked to determine the presence and features of the evaluated elements; discordances were resolved by a third reader (neuroradiologist with 22 years of experience).

To assess which of these pathological or non-pathological conditions are statistically more frequent in ADPKD than in general population, a control cohort, matched for age and sex distribution, has been retrospectively established. This control group was composed by all the consecutive MR with angiographic sequences performed at the Neuroradiological Department IRCCS Istituto delle Scienze Neurologiche in Bologna from 1 January 2017 to 31 December 2019 in non-ADPKD patients. To avoid selection bias, only the first MR requested by the general practitioner for nonspecific symptoms, such as headache, vertigo or trauma, has been included. Examinations performed as follow-up of known brain or vascular pathologies, or for presence of neurological symptoms as intracranial hypertension, motor or sensitive deficits, were excluded. Considering the rate of ADPKD patients with IA evaluated in our study and the expected incidence of IA in the general population (3%), a sample size of 300 subjects in the control cohort was considered sufficient to obtain statistically relevant results (alpha: 5% and 1‑beta: 80%) analyzing the same MR findings as in ADPKD patients. We also collected abdominal MR reporting the total kidney volume (TKV), in order to assess a possible relationship with the trend of the extrarenal disease.

Finally, demographic, laboratory, clinical, familial and genetic parameters have been collected to identify which patients are at higher risk of positive brain findings at MR screening, and to ascertain whether their pre-existing pathological or non-pathological conditions are significantly associated to ADPKD. The data were obtained from digital medical records. The study was conducted in accordance with the principles of the Declaration of Helsinki and was approved by the local ethics committee.

## Genetic Analysis

Genomic DNA was isolated from EDTA peripheral blood using the semi-automatic Maxwell 16 instrument (Promega Corporation, Madison, WI, USA).

The PKD1 (MIM#601313 HGNC:9008, RefSeq NM _001009944.2) entire coding region, including exon-intron boundaries (at least 50 bp) and most of the 5′ and 3′ untranslated regions were amplified in 8 long-range (LR) PCR using specific primers anchored on the rare mismatch sequencing between PKD1 and pseudogenes. We combined previously described primers [43–45] with primers designed by us and we optimized the PCR protocols. A LR-PCR method was validated also for PKD2 (MIM#173910 HGNC:9009, RefSeq NM_000297.3), using previously described primers [44]. For each patient, all 13 LR-PCR products (37.8 kb for PKD1 and 35.9 kb for PKD2) were pooled together in equimolar manner (50 pM each), the pools were then subjected to enzymatic digestion using Ion Xpress Plus Fragment Library Kit, in order to obtain about 200 bp fragments. Fragmented LR-PCRs were used to construct barcoded libraries using Ion Xpress barcode adapters following the manufacturer’s protocol. A single barcode was used for simultaneous analysis of both genes for each patient. Size selection of fragments was performed by E‑Gel Size Select II 2% agarose gel (Thermo Fisher Scientific).

## Statistical Analysis

Continuous data are presented as median or mean ± standard deviation (SD) and categorical variables as absolute numbers and percentage, as appropriate. Correlations were assessed using Pearson’s or Spearman’s method, for normally or non-normally distributed data, respectively. A χ^2^-test (Fisher’s exact test) was used for comparing categorical variables. First, we carried out a univariate regression logistic model for each parameter, to compare the MR findings rate in the two cohorts. Subsequently, a further univariate and regression logistic model was used to select the variables influencing the outcome of interest (MR findings statistically relevantly associated with ADPKD patients at previous analysis).

The parameters compared in the first analysis (ADPKD vs. non ADPKD patients) were: presence of IA, presence of multiple IAs, their maximum size and dome-to-neck ratio, presence of AC, other vascular lesions, anatomical arterial variants (considering separately the fetal variant of PComA and the other variants) and PHASES score.

In the second analysis, the following parameters were computed: sex, age, serum creatinine, eGFR, presence of arterial hypertension, therapy with RAAS blockers, smoking habit, genetic features and positive familiar history for ADPKD and IA/SAH.

Finally, a multivariate regression analysis including variables that remained significant was performed. A *p*-value < 0.05 was considered statistically significant. All the statistical analysis were performed using STATA statistical software, version 13 (StataCorp LP. College Station, TX, USA).

## Results

The whole ADPKD population of patients followed at our center in the period between January 2016 and December 2019 consisted of 156 patients. Among them, 125 underwent a brain MRI with angiographic sequences and were included in this study (70 women, 54%, mean age 46 ± 17 years).

The clinical features of our series are reported in Table 1. Particularly, mean serum creatinine was 1.2 ± 0.6 mg/dL, eGFR based on CKD-EPI was 76.6 ± 30.0 mL/min. As expected in an ADPKD population, most of our patients had arterial hypertension (84, 67.2%), and 76 (60.8%) were in treatment with RAAs blockers. Of the patients 21 (16.8%) were active smokers, 27 (21.6%) were former smokers, 77 (61.6%) never smoked. Genetic DNA sequencing for PKD1 and PKD2 was performed in 97 out of 156 (77.6%) subjects: 36 (28.8%) had a truncated PKD1 mutation, 36 (28.8%) had a non-truncated PKD1 mutation, 10 (8%) had a PKD2 mutation and 15 (12%) presented a negative DNA sequencing for PKD1 and PKD2 genes. In our population, 101 (80.8%) patients had a positive family history for ADPKD and 22 (17.6%) patients had a positive family history for IAs or SAH.

**Table 1:** Demographic and clinical features of the ADPKD patient series. Continuous variables are presented as mean ± standard deviation (SD) and categorical variables as absolute numbers and percentage

	ADPKD (*n* = 125)	Control cohort (*n* = 300)	*p*-value
*Sex (female)*	70 (56%)	195 (65%)	0.08
*Age (years)*	46.0 ± 17	47.3 ± 18	0.4
*Serum creatinine (mg/dL)*	1.2 ± 0.6	–	–
*eGFR (mL/min)*	76.6 ± 30.0	–	–
*Arterial hypertension*^a^	84 (67.2%)	139 (46.3%)	*0.001*
*RAAS blocker*	76 (60.8%)	98 (32.7%)	0.001
*Smoking habit*			
Active smokers	21 (16.8%)	69 (23%)	0.15
Former smokers	27 (21.6%)	74 (24.7%)	0.46
No smokers	77 (61.6%)	167 (55.7%)	0.26
*Genetic features*			
Truncant PKD1 mutation	36 (28.8%)	–	–
Non-truncant PKD1 mutation	36 (28.8%)		
PKD2 mutation	10 (8%)		
No mutations	15 (12%)		
NA	28 (22.4%)		
*Familiar history for ADPKD*			
Positive	101 (80.8%)	–	–
Negative	24 (19.2%)		
*Familiar history for IAs or SAH*			
Positive	22 (17.6%)	–	–
Negative	103 (82.4%)		

*ADPKD* adult polycystic kidney disease; *eGFR* estimated glomerular filtration rate; *IAs* intracranial aneurysms; *RAAS* renin-angiotensin-aldosterone-system; *SAH* subarachnoid hemorrhage, *NA* not available

^a^Arterial hypertension was considered when blood pressure > 140/90 mm Hg.

As reported in Table 2, brain MR revealed that 17 (13.6%) ADPKD patients had a IAs, multiple in 3 (17.6%) cases with a mean maximum diameter of 3.3 ± 1.4 mm and dome-to-neck ratio of 1.6 ± 0.6. The IAs were located as follows: ICA siphon (*n* : 10), middle cerebral artery (*n* : 2), anterior cerebral artery (*n* : 3), basilar apex (*n* : 1) 14 (11.2%) patients presented ACs, and 6 (4.8%) another kind of vascular lesion (mainly cavernomas). Moreover, we found that 23 (18.4%) patients showed at least 1 arterial anatomical variant, such as fenestrations, duplications, azygos vessels. The more common variant was represented by the fetal variant of PComA, which was detected in 12 cases (9.6%), while other variants (mainly fenestrations) were found in 13 patients (10.4%).

**Table 2:** MR findings in ADPKD patients and control cohort

	ADPKD (*n* = 125)	Control cohort (*n* = 300)	*p*-value
*Aneurysms*	17 (13.6%)	16 (5.3%)	*0.005*
*Multiple aneurysms*	3 (17.6%)	4 (25%)	0.62
*Maximum diameter*	3.3 ± 1.4	3.8 ± 1.9	0.48
*Dome-to-neck ratio*	1.6 ± 0.6	1.1 ± 0.9	0.48
*Mean PHASES score*	2.2 ± 1.9	1.9 ± 1.8	0.65
*Arachnoid cyst*	14 (11.2%)	6 (2%)	*<* *0.001*
*Arterial anatomical variants*	23 (18.4%)	33 (11%)	*0.04*
*Fetal PComA*	12 (9.6%)	26 (8.6%)	0.51
*Other (not fetal PComA) arterial anatomical variants*	13 (10.4%)	7 (2.3%)	*0.001*
*Other vascular lesions*	6 (4.8%)	10 (3.4%)	0.48

*ADPKD* adult polycystic kidney disease; *PComA* posterior communicating artery

Table 3 illustrates the results of the multivariate analysis of the variables potentially associated to a higher risk of IAs, but none reached statistical significance.

**Table 3:** Multivariate analysis among demographic, clinical and genetic factors and intracranial aneurysms in ADPKD patients. Data are presented as number (percentage) or medium ± standard deviation when adequate. The results of the multivariate are expressed as odds ratio and interquartile (IQ) interval

	Frequency	Odds ratio (IQ)	P‑value
*Sex (M/F)*	5 (29.4)/12(70.6)	0.49 (0.1–2.26)	0.36
*Age (years)*	49.0 ± 10.6	0.99 (0.89–1.1)	0.92
*Serum creatinine (mg/dL)*	1.3 ± 0.7	0.46 (0.03–6.58)	0.57
*eGFR (ml/min)*	65.5 ± 26.7	0.96 (0.9–1.03)	0.28
*Hypertension*	12 (70.6)	0.45 (0.03–7.27)	0.57
*RAAS blocker*	10 (58.8)	1.93 (0.17–21.84)	0.59
*Smoke*	4 (23.5)	0.77 (0.39–1.52)	0.45
*Genetic:*			
PKD1 t	3 (17.6)	1.04 (0.05–20.48)	0.98
PKD1 nt	10 (58.8)	7.33 (0.65–82.45)	0.11
PKD2	0 (0)	–	–
Negative	1 (5.9)	–	–
*Familial history for ADPKD*	12 (70.6)	4.98 (0.86–28.88)	0.07
*Familial history for IAs or SAH*	5 (29.4)	0.43 (0.09–2.11)	0.3

*ADPK* autosomal dominant polycystic kidney disease; *eGFR* estimated glomerular filtration rate; *F* female; *IAs* intracranial aneurysms; *M* male; *nt* not truncant; *SAH* subarachnoid hemorrhage; *RAAS* renin-angiotensin-aldosterone-system; *t* truncant

**Fig. 1:**
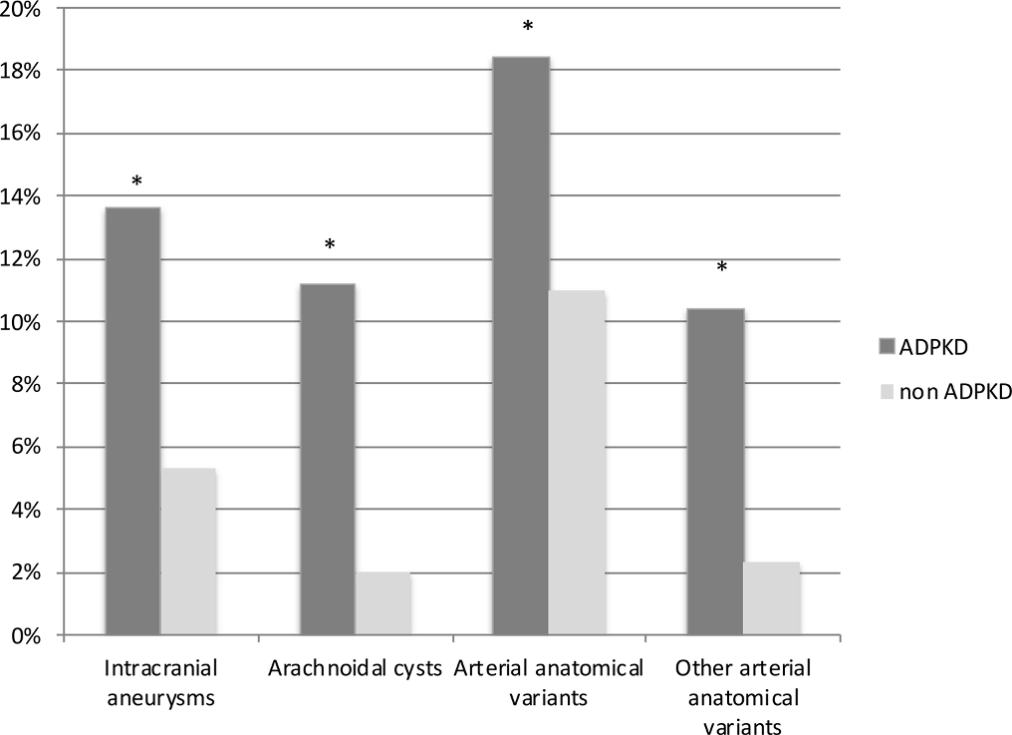
Differences in Brain MR findings between ADPKD and non-ADPKD population. The image shows that aneurysms, aracnoidal cysts, arterial anatomical variants and other arterial anatomical variants are increased in ADPKD population. All differences are statistically significant (* = *p* < 0.05). *ADPKD* Adult polycystic kidney disease

As expected, MR revealed a significantly higher incidence of IAs in ADPKD patients compared to the control cohort of 300 non-ADPKD subjects (13.6% vs*.* 5.3%, respectively, *p* : 0.005). Besides, also ACs (11.2% vs. 2%, *p* < 0.001) and arterial anatomical variants (18.4% vs. 11%, *p* = 0.04) were significantly more frequent in ADPKD patients than in controls. Among these variants, the most common, represented by the fetal variant of PComA, was similarly represented in the two groups, while the difference for other variants (mainly arterial fenestration) reached a level of significance (10.8% vs. 2.3%, *p* : 0.001) (see Fig. 1). Conversely, the two cohorts did not differ in terms of the number of multiple aneurysms and their mean size, dome-to-neck ratio, PHASES score and incidental findings of other vascular lesions (cavernomas).

We analyzed the features potentially associated to a higher risk of MR findings in the univariate analysis. In particular, no parameter was associated to an increased frequency of anatomical variants in general or non-fetal PComA anatomical variants. As expected in view of their congenital origin, no association of these inborn defects was observed with patients’ habits (smoke), treatment or acquired diseases (arterial hypertension). Interestingly, also ADPKD genetic profiles or positive family history of ADPKD did not result significantly correlated with the presence of these variants.

For similar reasons, non-inherited features (smoking habits, drug treatment or arterial hypertension), were also unrelated to ACs. Considering the genetic profiles, we found that these cysts were present only in patients with truncated or nontruncated mutations of PKD‑1 gene, although these results were not statistically significant. Likewise, no association of IAs was observed with the same non-inherited parameters, as well as age and gender. Significantly, in the subgroup of patients who experienced aneurysms, 10 (58.8%) patients had a non-truncant PKD1 mutation, 3 (17.6%) had a truncant PKD1 mutation, 1 (5.9%) carried a different mutation from PKD1 or PKD2, and the genetic analysis of 3 (17.6%) was unavailable. Taken together, 13 (74.2%) patients presented a PKD1 mutation while nobody presented a PKD2 mutation, although this finding did not meet statistical significance.

Most intracranial abnormalities were found in those subjects who already had a family history of ADPKD: 12 (70.6%) aneurysms, 12 (85.7%) AC, 20 (87%) arterial anatomical variant, 12 (80%) other anatomical variants.

When analyzing abdominal MR evaluating TKV of 81 ADPKD patients (1717 ± 1440.9 mL) we found no differences in the group presenting IAs (1682.6 ± 1416 mL vs. 1568.5 ± 1700.8 mL) or in the group presenting AC (1635.7 ± 1643.6 mL vs. 1687.1 ± 1405.3 mL).

## Discussion

In our retrospective case-control study on the MR brain findings in ADPKD patients, a significantly increased prevalence of IAs was found in ADPKD compared to general population (*p* < 0.005); however, IAs are not the only detected cerebral manifestation of the disease (*p* : 0.005). We also observed in ADPKD patients a significantly higher risk or developing an ACs (*p* < 0.001) and to present arterial variants (*p* : 0.04), particularly fenestrations (*p* : 0.001), which has been demonstrated to be an additional risk factor for the development of IAs [10]. Indeed, most of the patients in our population presenting IAs showed a negative family history for IAs.

The association of IAs and ADPKD has been known for many years. The reported prevalence of IAs in ADPKD is 4–5 times higher than in the general population, with an estimated range between 9% and 12% (vs. 2–3% in the general population) [7, 11–14]. In this study we compared for the first time two cohorts (ADPKD vs. non ADPKD) of subjects submitted to brain MR, finding a significant difference in the percentage of IAs between the two populations (13.6% vs. 5.3% respectively). As mentioned, the only characteristic clearly associated with the presence of IAs in the literature is a positive family history [8]. Indeed, a meta-analysis conducted by Zhou et al. [15] showed that in the case of a first-degree relative with IAs (ruptured or unruptured) or SAH, the risk of developing a IA is 2.3-fold increased in comparison to ADPKD patients without a positive familiar history. The authors also suggested that specific ethnic populations, such as patients from China, Japan or Europe, should be considered at elevated risk. Consequently, the KDIGO guidelines do not recommend brain MRI as a widespread screening method, suggesting instead to reserve it only for patients with positive family history [7].

However, Pirson et al. reported that ADPKD patients without a family history have a 5.9% prevalence of IAs [8]. A retrospective study by Xu et al. showed a higher prevalence of IAs (21.6%) in patients with a positive family history; nevertheless, the prevalence in patients with a negative family history is 11%, far higher than in general population [12]. Niemczyk et al. reported that the prevalence of IAs in their study was 16.9%, but they noticed that for older populations (> 45 years) the frequency increased to 22.4%. The authors did not find an increased prevalence in the subjects with a family history of IAs [3].

In our series, we found that 70.6% of IAs (12 cases) occurred in patients with negative family history. These results are in line with the a previous large retrospective study of Sanchis et al. [5] on 812 ADPKD patients visiting the Mayo Clinic between 1989 and 2017 who underwent screening MRA, where a prevalence of 9% for IAs (94 cases in 75 patients which presents aneurysms) was reported. The IAs were detected in 47 of 652 (7%) patients without family history; in this population, 67 of 75 patients (89%) were older than 40 years. Our results are also consistent with the findings by Niemczyk et al. who proposed to screen all patients with ADPKD older than 45 years, based on the elevated number of patients in this category presenting aneurysms [3]. According to our data, we would support a new trend to extend the MR screening also to patients without a family history of IAs.

Several studies sought to understand the link between genetic mutations and the development of IAs. Aneurysm formation might depend on focal, somatic and random loss of the wild-type PKD allele in the vascular tissue, as it has been demonstrated in the cyst lining epithelium of the kidneys and liver [11, 13, 16]. A role has been suggested for polycystin complex as a pressure sensor in the vasculature [17]. There is a large body of evidence to suggest a direct involvement of the two proteins (Polycystin 1 and 2) in the development and maintenance of the myoelastic arterial wall of the small arteries in the brain and the development of IAs in ADPKD patients [17–19].

Although this finding did not meet statistical significance, in our population we observed an increased rate of IAs in patients with non-truncated PKD‑1 mutation (10 cases, 58.8%). Taken together, we reported 13 (76.5%) PKD1 mutations vs. 0 (0%) PKD2 mutations. These results, based on the available genetic tests (72.8% of patients), are in line with the current literature [20–22]; however, further studies with larger sample sizes are needed to fully elucidate the association between genetic mutations and IAs. As for arterial anatomical variants we found that fenestrations, duplications or azygos variants showed a higher incidence in ADPKD patients. A recent meta-analysis suggested that fenestrations might expose to an increased susceptibility of developing IAs [10]. Actually, aneurysm formation is favored by the turbulent flow created by defects in the tunica media, as the ones between the proximal and distal edges of a fenestrated arterial segment [10]. Similarly, other arterial variants, such as duplications azygos, may induce turbulent flow, favoring aneurysm formation [23]. Therefore, we can speculate that ADPKD might promote aneurysm formation onset by a double mechanism: on one hand through the arterial wall modifications on a genetic basis and on the other, through the alterations of the arterial flow, due to the presence of anatomical variants that could increase the risk of aneurysm formation.

The overall rupture rate of IAs in ADPKD patients is 5 times higher than in general population. The effectiveness of presymptomatic screening and interventions to prevent aneurysmal rupture depends on many factors: the prevalence of intracranial aneurysms, the availability of screening procedures, the risk of rupture with medical and antihypertensive therapy only, comorbidities, mortality associated with surgical or neuroradiological procedures of the aneurysm and risk of de novo aneurysm development and rupture [9, 24–26]. Nurmonen et al. tried to analyze the association of ADPKD with ruptured and unruptured IAs in a Finnish population (Kuopio and Finnish nationwide registries). The study pointed out that the median age of aneurysmal SAH for patients with ADPKD was significantly lower than in general IAs population; the number of ruptured IAs was significantly smaller than in general population, as for de novo IAs formation [27].

In our investigation, the size and the dome-to-neck ratio of IAs in ADPKD patients did not differ significantly, as well as the mean age; however, it has been demonstrated that the risk of an IA depends also on its enlargement rate [28]. Among our series, 17.6% of cases enlarged at follow-up, suggesting that regardless of their size, IAs may have a more aggressive behavior in ADPKD than in general population. Of course, the short observation time in our cohort prevents us to draw any firm conclusion about this issue.

ACs are collections of cerebrospinal fluid covered by a thin membrane that is continuous with the normal surrounding arachnoid; this membrane consists of arachnoid cells and collagen fibers in the extracellular matrix, considered to be as developmental abnormalities of the arachnoid. These may be manifestations of the underlying defect of extracellular matrix in ADPKD [29]. The natural history of ACs is variable but most of them remain stable throughout life and rarely lead to complications [26, 30, 31]. The prevalence of ACs in the general population varies from 1.1% to 2.3% [31, 32] amounting to 5.2–12.8% in patients with ADPKD [13, 29, 33, 34]. Even if most of the cysts are usually asymptomatic, symptomatic cysts can be associated with mental retardation, seizures, cerebral hemorrhage and focal neurological deficits [35–37]. Similarly to the general population, the main site of cystic lesions was the middle, followed by the posterior cranial fossa. Cyst size was variable, ranging from very small to bizarrely shaped large cysts encompassing major parts of the hemicranium. The changes in cyst volume seem to be unpredictable. In line with our findings, Schievink et al. reported increased prevalence of arachnoid intracranial cysts in a cohort of ADPKD patients compared to the general population (8.1% vs. 0.8%), although the study was focused on the correlation with polycystic liver disease (PLD). The authors found PLD in 17 (85%) out of the 20 patients with ACs, but only 119 (52.4%) of the 227 without [29].

Brain MR screening highlighted many vascular abnormalities and intracranial cysts, depicting an intracranial situation highly suggestive of a systemic disorder. The ADPKD patients with or without intracranial arachnoid cysts did not present any significant difference in terms of clinical features.

Total kidney volume (TKV) is a well-established prognostic biomarker of renal function decline and progression to end stage renal disease (ESRD) in patients with ADPKD [33, 38–40]. The TKV can be used in combination with age and eGFR as an additional predictor to identify the patients most likely to experience renal dysfunction. The CRISP study demonstrated that PKD1 mutations are associated with earlier progression to ESRD, larger TKV and greater cysts burden regardless of the age [41, 42]. Here, we tried to evaluate if there could be a correlation between TKV and the prevalence of IAs or intracranial abnormalities in order to detect patients with a potentially more severe prognosis. In our population, no differences were found between ADPKD patients presenting IAs and those who did not. To the best of our knowledge, no associations have been previously described on the presence of IAs or other intracranial abnormalities with TKV, and therefore our data are in line with literature. This suggest that even if ADPKD is a systemic disorder, different mechanisms can play a role in various aspects of disease progression.

## Limitations

The main limitations of the study are represented by the monocentric and retrospective nature and the limited sample size.

## Conclusion

In our study we found a higher incidence of IAs in ADPKD than general population, as well as ACs and arterial variants, which could in some cases be a trigger for the development of IAs. In the majority of patients with IAs, a PKD1 mutation was identified. Moreover, a significantly increased number of alterations was observed even in those patients without a family history of IAs or SAH. No other clinical, laboratory or instrumental conditions have been associated with IAs in ADPKD patients. A presymptomatic screening of all patients with ADPKD should be considered in order to detect intracranial findings, thus representing a helpful decision-making tool to achieve an optimal patient management.

## Abbreviations

AC: Arachnoid cysts
ADPKD: Adult polycystic kidney disease
BP: Blood pressure
CKD: Chronic kidney disease
CKD-EPI: Chronic kidney disease epidemiology collaboration
DWI: Diffusion weighted imaging
eGFR: Estimated glomerular filtration rate
ESRD: End stage renal disease
FLAIR: Fluid attenuated inversion recovery
FSE: Fast spin echo
IAs: Intracranial aneurysms
ICA: Internal carotid artery
KDIGO: Kidney disease improving global outcomes
LR: Long range
MR: Magnetic resonance
MRA: Magnetic resonance angiogram
PComA: Posterior communicating artery
PCR: Polymerase chain reaction
PLD: Polycystic liver disease
RAAs: Renin-angiotensin-aldosterone-system
SAH: Subarachnoid hemorrhage
SPIR: Spectral presaturation with inversion recovery
SWI: Susceptibility weighted imaging
TOF: Time of flight
TKV: Total kidney volume
